# DXD Motif-Dependent and -Independent Effects of the *Chlamydia trachomatis* Cytotoxin CT166

**DOI:** 10.3390/toxins7020621

**Published:** 2015-02-17

**Authors:** Miriam Bothe, Pavel Dutow, Andreas Pich, Harald Genth, Andreas Klos

**Affiliations:** 1 Institute for Medical Microbiology and Hospital Epidemiology, Hannover Medical School, Carl-Neuberg-Str. 1, D-30625 Hannover, Germany; E-Mails: bothe.miriam@mh-hannover.de (M.B.); dutow.pavel@mh-hannover.de (P.D.); klos.andreas@mh-hannover.de (A.K.); 2 Institute for Toxicology, Hannover Medical School, Carl-Neuberg-Str. 1, D-30625 Hannover, Germany; E-Mail: pich.andreas@mh-hannover.de

**Keywords:** chlamydia, glycosyltransferase, *Clostridium difficile* toxin B, *Clostridium sordellii* lethal toxin, Ras, Rho

## Abstract

The Gram-negative, intracellular bacterium *Chlamydia trachomatis* causes acute and chronic urogenital tract infection, potentially leading to infertility and ectopic pregnancy. The only partially characterized cytotoxin CT166 of serovar D exhibits a DXD motif, which is important for the enzymatic activity of many bacterial and mammalian type A glycosyltransferases, leading to the hypothesis that CT166 possess glycosyltransferase activity. CT166-expressing HeLa cells exhibit actin reorganization, including cell rounding, which has been attributed to the inhibition of the Rho-GTPases Rac/Cdc42. Exploiting the glycosylation-sensitive Ras(27H5) antibody, we here show that CT166 induces an epitope change in Ras, resulting in inhibited ERK and PI3K signaling and delayed cell cycle progression. Consistent with the hypothesis that these effects strictly depend on the DXD motif, CT166 with the mutated DXD motif causes neither Ras-ERK inhibition nor delayed cell cycle progression. In contrast, CT166 with the mutated DXD motif is still capable of inhibiting cell migration, suggesting that CT166 with the mutated DXD motif cannot be regarded as inactive in any case. Taken together, CT166 affects various fundamental cellular processes, strongly suggesting its importance for the intracellular survival of chlamydia.

## 1. Introduction

The DXD motif is a short, conserved motif found in many families of bacterial and mammalian type A glycosyltransferases [1]. DXD-containing glycosyltransferases, which exploit nucleoside diphosphate sugars as donors, transfer a range of different sugars to other sugars, phosphates and proteins. The best characterized families of DXD-containing bacterial glycosyltransferases are the *Legionella pneumophila* glucosyltransferases (Lgt1-3) and the large clostridial glucosylating cytotoxins (LCGTs). LCGTs enter their mammalian target cells by receptor-mediated endocytosis and mono-*O*-glycosylate small GTPases of the Rho- and Ras-subfamilies at pivotal threonine residues [2,3,4]. The LCGT family encompasses toxin A (TcdA) and toxin B (TcdB) from *Clostridium difficile*, the lethal (TcsL) and the hemorrhagic toxin (TcsH) from *Clostridium sordellii*, the alpha-toxin (TcnA) from *Clostridium novyi*, as well as the *Clostridium perfringens* large cytotoxin (TpeL) [5]. Mutation of both aspartic acids into any other amino acid have been reported to strongly reduce the enzymatic activity of DXD-containing clostridial glycosyltransferases [6,7,8]. The *Legionella* Lgt1-3 mono-*O*-glucosylate the eukaryotic elongation factor EF1A at a specific serine residue. Upon direct contact of *Legionella* with the mammalian target cells, Lgt2 and Lgt3 are secreted into the cytosol by the type IV secretion system (T4SS) [9,10]. Putative bacterial glycosyltransferases that contain a DXD motif have further been found in *Escherichia coli*, *Citrobacter rodentium*, *Photobacterium profundum*, *Pseudomonas fluorescens* and *Chlamydia* spp.

*Chlamydia trachomatis* (*Ctr*) is an obligate intracellular, Gram-negative bacterium of which different serovars are described. Serovars A–C cause the infectious eye disease, trachoma, the world’s leading cause of preventable blindness, whereas serovars D–K cause mainly acute and chronic inflammatory disease of the urogenital tract that can lead to infertility or ectopic pregnancy [11]. Furthermore, serovars L1–L3 cause lymphogranuloma venereum (LGV), a sexually-transmitted urogenital infection (often found in males having sex with males) that affects additionally the inguinal lymph nodes. *Chlamydiae* have a special biphasic productive cycle: infectious, but metabolically-inactive elementary bodies (EBs) enter the host cell, where they differentiate into metabolically-active reticulate bodies (RBs). Inside host-derived inclusions (small, membrane-bound compartments), the RBs multiply by binary fission. After approximately 20 h, they differentiate into a new generation of infectious EBs, which are finally released by host cell lysis or extrusion. In one genomic region of high variability, called the “plasticity zone”, an open reading frame (ORF) of 1917 bp, *CT166*, is present in *Chlamydia trachomatis* serovar D strain UW3 (*Ctr* D/UW3). On the protein level, CT166 exhibits high similarity with the *N*-terminal glycosyltransferase domain of LCGTs, including the DXD motif [2,12,13]. In *C. trachomatis* serovar L2 strain 434 (*Ctr* L_2_/434), no ORF with such sequence similarity is found. However, an unusual LGV-causing strain, termed *Ctr* L_2_c, has recently been described as a recombinant of L2 and D, exhibiting the complete *CT166* gene locus [14]. The putative glycosyltransferase CT166 is pre-formed in the EBs and found during the first 60 min in HeLa cells that were infected with high multiplicities of infection (MOI) of *Ctr* D/UW3 [2,12,13,15]. To directly investigate the role of CT166, it would have been helpful to generate *Ctr* D/UW3 lacking the functional ORF of CT166. However, the generation of such mutants in *Chlamydia* is still difficult and has not yet been successful in our hands. Instead, recently-established HeLa cell lines expressing CT166-wt and CT166-DA415A.D417A (CT166-mut) in a tetracycline-inducible vector (HeLa-CT166-wt or HeLa-CT166-mut cells) served for the continuation of the functional phenotypic characterization of CT166 [13]. Consistent with observations upon high MOI infection of HeLa cells with *Ctr* D/UW3, HeLa-CT166-wt cells exhibit actin reorganization, including a loss of cell spreading (cell rounding) [12], which has been attributed to the inhibition of the Rho-GTPase Rac1 [13]. Rac1 from HeLa-CT166-wt cells is not detected by Rac1(mAb102), an antibody incapable of detecting Rac/Cdc42 mono-*O*-glucosylated at Thr-35 by LCGTs [13,16,17].

In this study, we show that (H/K/N)Ras from HeLa-CT166-wt cells is not detected by Ras(mAb27H5), an antibody incapable of detecting (H/K/N)Ras mono-*O*-glucosylated at Thr-35 by TcsL [5,18]. This observation strongly suggests that CT166 induces an epitope change, not only in Rac/Cdc42, but also in (H/K/N)Ras. Furthermore, we are continuing the phenotypic characterization of CT166 with emphasis on its cell cycle effects. We here provide evidence on inhibited Ras-ERK signaling in HeLa-CT166-wt cells. HeLa-CT166-wt cells exhibit a delayed G1-S transition and diminished cell proliferation. Additionally, migration of both CT166-wt-, as well as CT166-mut-expressing cells is reduced.

## 2. Results and Discussion

### 2.1. Inhibition of Ras-ERK Signaling in Ctr D/UW3-Infected HeLa Cells

**Figure 1:**
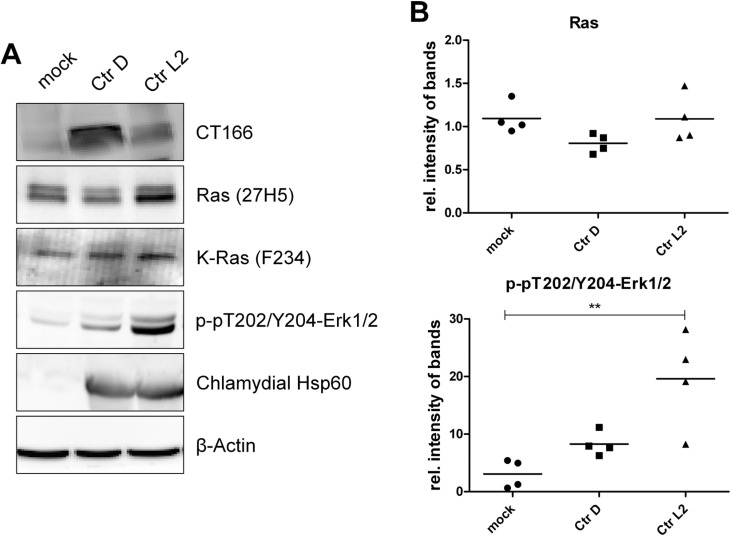
Changes in protein levels in HeLa cells infected with *Chlamydia trachomatis* serovar D strain UW3 (*Ctr* D/UW3) and L_2_/434. TRex™-HeLa cells were infected with either *Ctr* L_2_/434 or *Ctr* D/UW3 (MOI of five) or they were mock infected for 48 h. Cells were lysed, and the levels of the indicated proteins were analyzed by Western blot. Representative Western blots (**A**) and signal intensities of a minimum of four independent experiments are presented as relative (rel.) individual signal intensities and as means (**B**). For statistical analysis, one-way ANOVA and Tukey’s multiple comparison test were used (* indicates statistically-significant differences: ** *p* ≤ 0.01).

HeLa cells were infected with *Ctr* D/UW3 and *Ctr* L_2_/434 at an MOI of five. The level of chlamydial heat shock protein 60 (Hsp60) strongly increased in the host cells, confirming effective infection (Figure 1A). Chlamydia caused an increased level of pT202/pY204-p44/42MAPkinase (ERK1/2), indicative of ERK activation. This is a well-described anti-apoptotic response of host cells to infection with chlamydia [19,20,21]. Remarkably, ERK1/2 activation was more pronounced in *Ctr* L_2_/434-infected than in *Ctr* D/UW3-infected HeLa cells (Figure 1B). *Ctr* D/UW3 (not *Ctr* L_2_/434) produces the DXD motif containing the CT166 cytotoxin, which has formerly been shown to inactivate small GTPases of the Rho subfamily [13] (Figure 1). The canonical pathway stimulating ERK1/2 by growth factors and their receptor tyrosine kinases includes (H/K/N)Ras.

Reduced ERK1/2 activation in *Ctr* D/UW3-infected cells leads to the hypothesis that CT166 (besides Rho-GTPases) also induces an epitope change of (H/K/N)Ras. Exploiting the Ras(mAb27H5) antibody incapable of detecting (H/K/N)Ras once being mono-*O*-glucosylated at Thr-35 [18], the cellular levels of (H/K/N)Ras were decreased in *Ctr* D/UW3-infected cells as compared with either mock- or *Ctr* L_2_/434-infected cells (Figure 1B). The cellular level of exemplarily K-Ras was comparable in *Ctr* L_2_/434- or mock-infected cells, as analyzed exploiting the K-Ras(mAbF234) antibody. The latter antibody detects K-Ras independently of mono-*O*-glucosylation at Thr-35 [18]. Thus, reduced Ras detection by the Ras(27H5) antibody did not result from Ras degradation. These observations suggest that impaired ERK1/2 activation in *Ctr* D/UW3-infected HeLa cells results from an inactivating epitope change in (H/K/N)Ras.

### 2.2. CT166-Mediated Inhibition of Ras-ERK and PI3K/Akt Signaling

**Figure 2:**
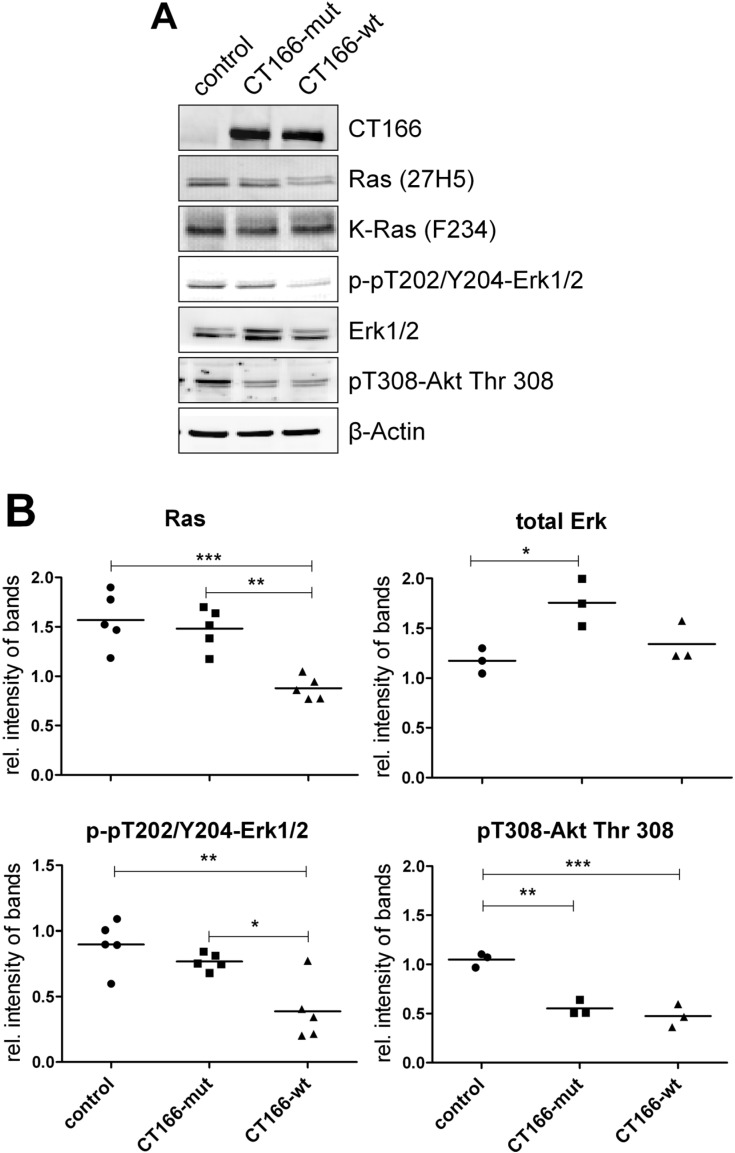
Expression of CT166-wt or CT166-mut reduces the level of several proteins. To augment the expression of the chlamydial proteins, HeLa-control, HeLa-CT166-mut and HeLa-CT166-wt cells were incubated for 24 h in the presence of tetracycline (1 µg/mL). Cells were lysed, and the levels of the indicated protein were analyzed by Western blot. Representative Western blots (**A**) and quantified signal intensities (including the means) (**B**) of a minimum of three independent experiments are presented. To calculate the relative (rel.) intensity of bands, the signal levels of equally-treated, unaltered TRex™-HeLa cells were defined as 1.0 for each antibody. For statistical analysis, one-way ANOVA and Tukey’s multiple comparison test were used (* indicates statistically-significant differences: * *p* ≤ 0.05; ** *p* ≤ 0.01; *** *p* ≤ 0.001).

To further show that CT166 modifies Ras-GTPases, HeLa cells stably expressing CT166-wt (HeLa-CT166-wt cells) or CT166-mut (HeLa-CT166-mut cells) in a tetracycline-inducible vector were used [13]. Upon treatment with tetracycline for 24 h, the cellular levels of CT166-wt and CT166-mut were strongly increased to comparable amounts in the corresponding HeLa cells (Figure 2A). The cellular levels of (H/K/N)Ras were markedly decreased in HeLa-CT166-wt cells (but not in HeLa-CT166-mut cells), as analyzed exploiting the glucosylation-sensitive Ras(mAb27H5) antibody (Figure 2B). In contrast, the cellular level of total K-Ras was comparable in HeLa-CT166-wt, in HeLa-CT166-mut and in HeLa-control cells, based on the analysis with the K-Ras(mAbF234) antibody. Reduced detection by Ras(27H5) antibody pointed to an epitope change of Ras not due to degradation. Besides Rho-GTPases, CT166 seems to also induce an epitope change in Ras-GTPases.

Upon treatment of cultured cells with Ras/Rac-glucosylating toxins, including *Clostridium sordellii* lethal toxin (TcsL) or *Clostridium perfringens* large cytotoxin (TcpL), ERK1/2, as well as Akt have been found to be dephosphorylated, indicating ERK and Akt inactivation [22,23]. In HeLa-CT166-wt cells, the levels of pT202/Y204-ERK1/2, as well as pT308-Akt were strongly reduced (Figure 2B). Interestingly, the level of pT308-Akt was also reduced in HeLa-CT166-mut cells, suggesting that CT166-mut is not inactive. In regard to ERK and Akt inactivation, the CT166-wt thus phenocopies the effects of the Ras/Rac-glucosylating TcsL and TpeL, supporting the hypothesis that CT166 DXD-dependently catalyzes a not yet identified epitope change in Ras. Moreover, impaired ERK1/2 activation in *Ctr* D/UW3-infected cells correlates with CT166-induced Ras inactivation.

**Figure 3:**
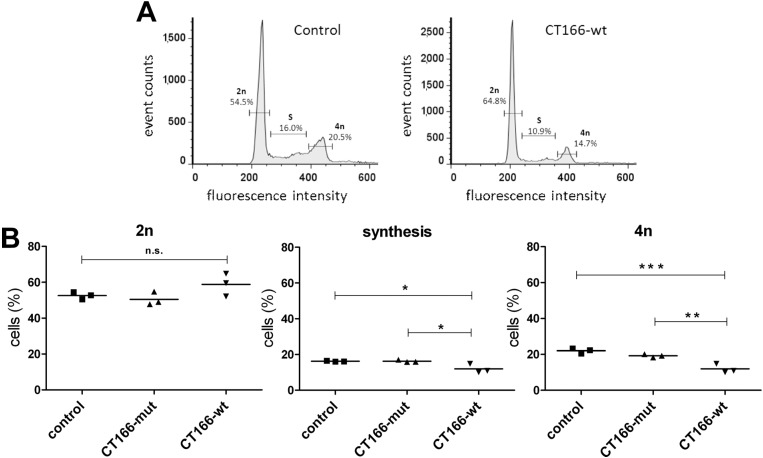
CT166 expression delays G1-S transition of cell cycle. HeLa-control, HeLa-CT166-mut and HeLa-CT166-wt cells were incubated in the presence of tetracycline (1 µg/mL) for 24 h. Cells were fixed, and the DNA was stained with propidium iodide. The DNA content was analyzed by flow cytometry. Depicted are two exemplary histograms for HeLa-control and HeLa-CT166-wt cells representing DNA-dependent fluorescence intensity *versus* cell frequency within 20,000 events analyzed by FACS (**A**). The same gates for the determination of the percentages of cells being in the 2n, synthesis (S) or 4n stages of the cell cycle, which were first set for the HeLa-control, were also applied for the other two cell lines. The results, *i.e.*, the percentage of cells being in the indicated different stages of the cell cycle of three independent FACS experiments, each analyzing the three different cell lines in parallel and, in addition, their means, are depicted (**B**). One-way ANOVA and Tukey’s multiple comparison test were used for statistical evaluation (* indicates statistically-significant differences: * *p* ≤ 0.05; ** *p* ≤ 0.01; *** *p* ≤ 0.001; n.s. = not significant).

Next, the hypothesis was addressed if CT166-mediated inhibition of Ras-ERK and of PI3K-Akt signaling results in delayed cell cycle progression. This hypothesis is based on the rationale that both ERK and PI3K regulate cell cycle progression at the G1-S, as well as the G2-M boundaries [24]. CT166-mediated inhibition of ERK and PI3K, in turn, might result in delayed cell cycle progression and reduced cell proliferation. Cell cycle progression was determined by FACS analysis of propidium iodide-stained cells. In particular, the number of HeLa-CT166-wt cells in the S-phase and the G2-M (4n)-phase were significantly diminished (as compared with either HeLa-CT166-mut or HeLa-control cells) (Figure 3B), indicating delayed G1-S transition. Delayed G1-S transition is a major cause of an increased doubling time. HeLa-control and HeLa-CT166-mut cells exhibited comparable doubling times of about 35 h, as estimated by dividing the natural logarithm of two by the exponent of growth. The proliferation of HeLa-CT166-wt cells was strongly reduced, as they exhibit a doubling time of about 100 h. Consistent with delayed G1-S transition, CT166 DXD motif-dependently causes delayed cell proliferation. In sum, the yet not identified covalent modification of (H/K/N)Ras correlates with the inhibition of the distal effector proteins ERK1/2 and Akt (Figure 2B). ERK1/2 inhibition (and most likely, subsequent Cyclin D1 suppression) seems to be responsible for the delayed G1-S transition and reduced proliferation of HeLa-CT166-wt cells. In HeLa-CT166-mut cells, Ras-ERK signaling is seemingly not affected, which correlates with the observation that the proliferation of HeLa-CT166-mut and HeLa-control cells are comparable. Thus, CT166-induced inhibition of proliferation depends on the DXD motif.

### 2.3. Formation of Multinucleated CT166-Expressing HeLa Cells

**Figure 4:**
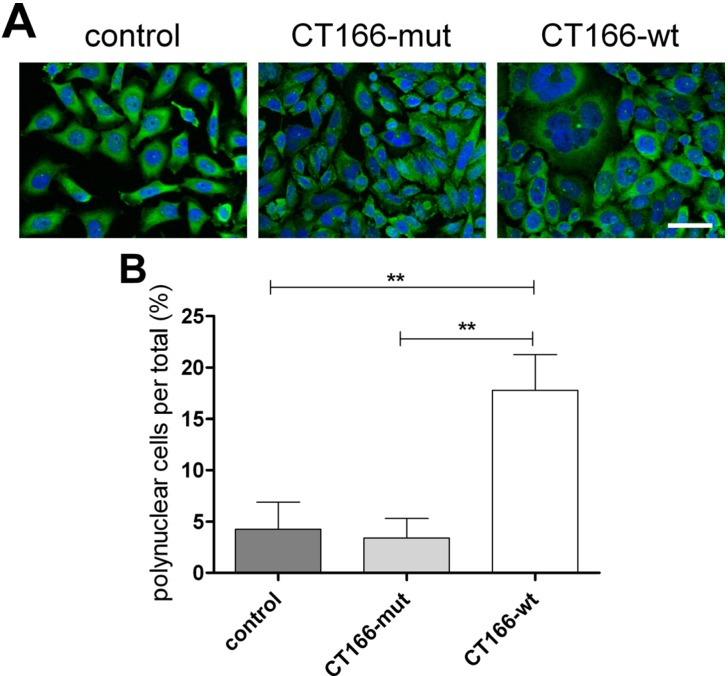
CT166-wt induces the formation of multinucleated cells. HeLa-control, HeLa-CT166-mut and HeLa-CT166-wt cells were treated with tetracycline (1 µg/mL for 24 h) for protein expression, and then, the microtubule network and cell nuclei were stained (scale bar represents 50 µm). Representative fluorescence images (**A**) and the percentage of multinuclear cells ± SD (**B**) of three independent experiments are demonstrated (using the identical magnification factor). For statistics, one-way ANOVA and Tukey’s multiple comparison test were applied (* indicates statistically-significant differences: ** *p* ≤ 0.01; n.s. = not significant).

The final steps of mitosis include contractile ring formation in cytokinesis and abscission. Formation of the contractile actin-myosin ring strictly depends on the activity of RhoA [25]. Multinucleation indicating the inhibition of cell cytokinesis has been reported in proliferating cells in response to bacterial protein toxins that covalently modify RhoA, including the Rho ADP-ribosylating *Clostridium botulinum* exoenzyme C3 [26], the RhoA glucosylating *Clostridium difficile* toxins TcdA and TcdB [27,28] or RhoA deamidating the cytotoxic necrotizing factor from *Yersinia pseudotuberculosis* [29,30]. In either case, the covalent RhoA modification blocks the contractile ring formation of cytokinesis without causing an arrest of cell cycle progression. The cells thus skip cell division and re-enter the cell cycle as bi- or multi-nucleated cells. Inhibition of host cell cytokinesis has also been reported during *Ctr* infection of various cell lines with different serovars (including *Ctr* D/UW3 and *Ctr* L_2_/434) [31]. Furthermore, transient transfection and overexpression of the chlamydial effector protein CT223, which is located in the inclusion membrane of *Ctr* D/UW2 and *Ctr* L_2_/434, is also reported to inhibit host cell cytokinesis and to cause multinucleation [32]. To check whether the expression of CT166 causes multinucleation as well, nuclei of HeLa-control, HeLa-CT166-mut and HeLa-CT166-wt cells were stained with DAPI. Indeed, ~15% of HeLa-CT166-wt cells are bi- or multi-nucleated (Figure 4B), while bi- or multi-nucleation was hardly (<5%) observed in HeLa-CT166-mut or HeLa-control cells. Although HeLa-CT166-wt cells (albeit delayed) progress through several cell cycles, they skip cell division, resulting in multinucleated cells. CT166-induced formation of multinucleated cells thus depends on the DXD motif. The formation of bi- and multi-nucleated cells strongly suggests that CT166 (at least) contributes to the *Ctr* D/UW3-induced inhibition of host cell cytokinesis during infection.

**Figure 5:**
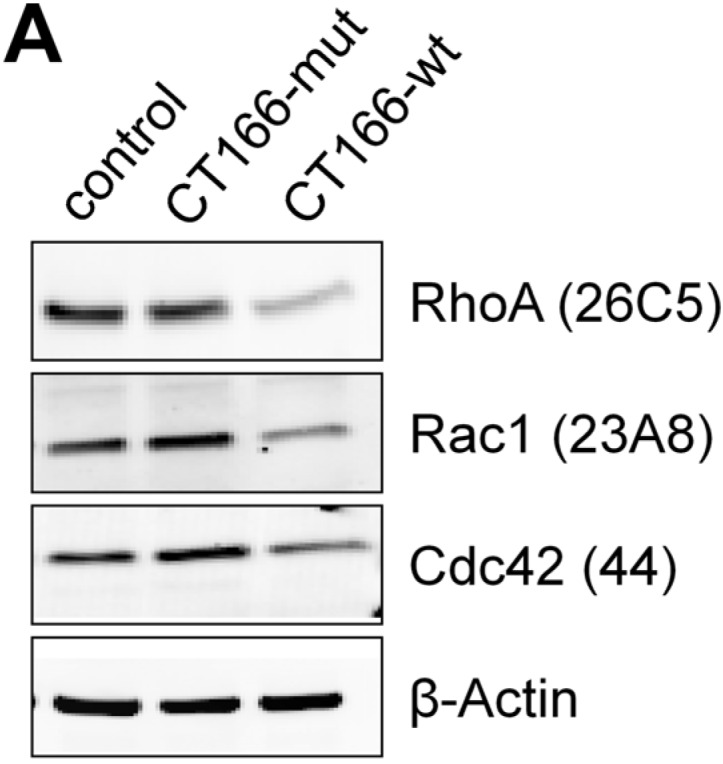

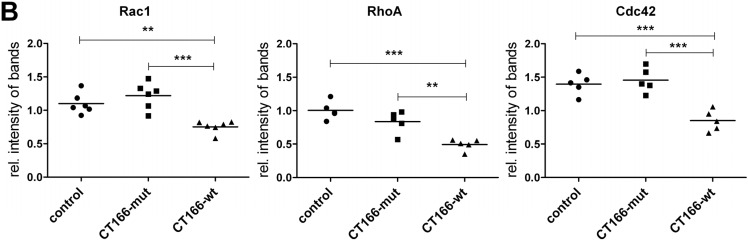
Reduced levels of RhoA, Rac1 and Cdc42 in HeLa-CT166-wt cells. Levels of RhoA, Rac1 and Cdc42 were analyzed in tetracycline-induced (1 µg/mL for 24 h) HeLa-control, HeLa-CT166-mut and HeLa-CT166-wt cells by Western blot. Representative Western blots (**A**) and quantified signal intensities (including means) (**B**) of a minimum of five independent experiments are depicted. To calculate the relative (rel.) intensity of bands, the signal levels of equally-treated, unaltered TRex™-HeLa cells were defined as 1.0 for each antibody. For statistical analysis, one-way ANOVA and Tukey’s multiple comparison test were used (* indicates statistically-significant differences: ** *p* ≤ 0.01; *** *p* ≤ 0.001).

Moreover, the observed multinucleation suggests that CT166 affects RhoA. In fact, the cellular level of RhoA (comparable to Rac1 and Cdc42) was clearly reduced in HeLa-CT166-wt (not HeLa-CT166-mut or HeLa-control) cells (Figure 5B). Thus, the not yet identified CT166-catalyzed modification of Rho-GTPases seems to mediate high susceptibility to proteasomal degradation, a feature known from ADP-ribosylated, glucosylated and deamidated Rho-GTPases [17,33,34]. Clearly, reduced cellular levels of RhoA correlate with inhibited contractile ring formation and subsequent multinucleation within a subpopulation of HeLa-CT166-wt cells.

### 2.4. Reduced Migration of CT166-Expressing HeLa Cells

**Figure 6:**
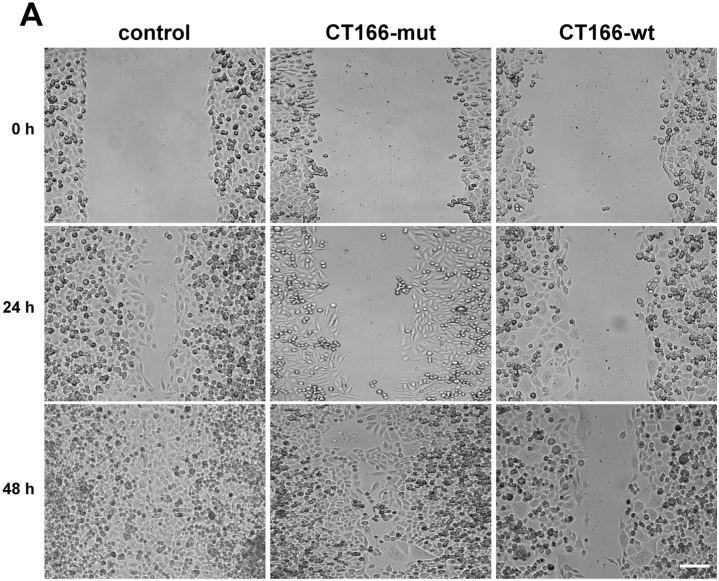

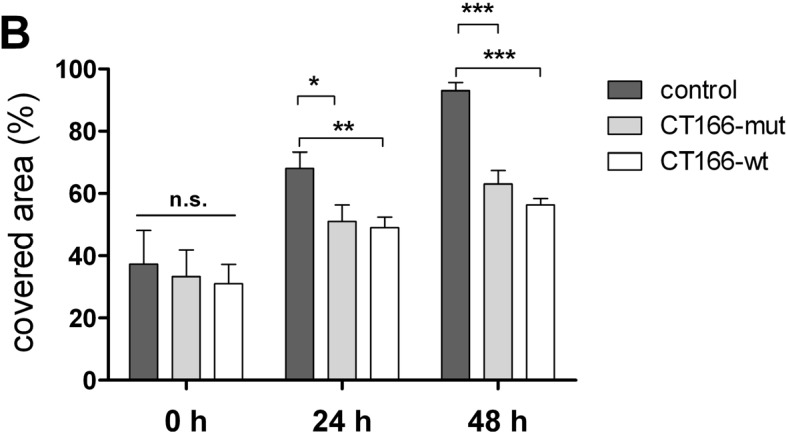
CT166 reduces the migration of HeLa-cells independently of the DXD motif. Confluent, tetracycline-induced (1 µg/mL for 24 h) HeLa-control, HeLa-CT166-mut and HeLa-CT166-wt monolayers were scratched and analyzed by phase contrast microscopy (**A**) after 24h and 48 h during 2 mM thymidine treatment (scale bar represents 200 µm). The determined mean ± SD of the covered area (in phase contrast images) of three independent experiments is depicted (**B**). For statistical evaluation, two-way ANOVA and the Bonferroni post-test were performed (* indicates statistically-significant differences: * *p* ≤ 0.05; ** *p* ≤ 0.01; *** *p* ≤ 0.001; n.s. = not significant).

Chlamydia causes fragmentation of the Golgi compartment to ensure its intracellular reproduction [35]. Fragmented Golgi (together with the lost orientation of the microtubule organizing center) is responsible for the reduced migration of *Ctr* L_2_/434-infected HeLa cells [36]. Rho-GTPases are the key regulators of cell migration, which have been suggested to be inactivated by CT166 [13]. This leads to the hypothesis that CT166 contributes to inhibited migration of *Ctr* D/UW3-infected HeLa cells. To check if CT166 decreases cell migration, reconstitution of confluent HeLa-CT166-wt, HeLa-CT166-mut and HeLa-control cells was analyzed upon mechanical injury (“scratch assay”) (Figure 6A). To exclude any influence of a differing rate in cell proliferation, it was inhibited by thymidine arresting the migrating cells in the pre-S-phase [37]. Migration of HeLa-control cells resulted in complete reconstitution of the monolayer upon 48 h (Figure 6B). In contrast, the migration of either HeLa-CT166-wt or HeLa-CT166-mut cells was strongly reduced, as only partial reconstitution of the monolayer was observed upon 48 h. This demonstrates that overexpression of CT166 (even with a mutated DXD motif) is sufficient for blocking HeLa cell migration. In fact, ectopically-expressed CT166-mut is not inactive, as it has formerly been shown to bind cellular Rho-GTPases, in terms of cellular Rho-GTPases being protected from TcdB-catalyzed glucosylation in Hela-CT166-mut cells [13]. Furthermore, binding of Rho-GTPases by ectopically-expressed CT166-mut is likely causative of the partially rounded morphology of HeLa-CT166-mut cells [13]. Against this background, it appears to be plausible that CT166-mut is capable of reducing cell migration (Figure 6B). Thereby, binding to Rho-GTPases is likely mediated by domains distinct from the (extended) DXD motif, because (as deduced from the TcdB structure) the DXD motif is involved in nucleotide-hexose, rather than in GTPase substrate binding [38]. On the other hand, the absence of multinucleated HeLa-CT166-mut cells shows that the binding capacity of ectopically-expressed CT166-mut is apparently not sufficient for blocking RhoA to the extent required for the inhibition of contractile ring formation. The capability of ectopically-expressed DXD-mutated enzymes seemingly results in substrate binding and, thereby, in partial inactivation. The latter consideration is of general importance, because the mutation of the DXD motif is an often applied method to shut down the activity of DXD-harboring enzymes [6,39].

In conclusion, CT166 is capable of influencing several cellular functions within the *Ctr* D/UW3-infected host cell processes. This includes actin dynamics, cell migration, cell cycle progression and proliferation. Ectopic expression of CT166 allows the identification of new CT166 functions and addressing the question of whether the CT166 effects depend on the DXD motif. However, one should keep in mind that the CT166 effects are most likely much more restricted during chlamydial infection, where CT166 might be transiently expressed to lower levels. Ectopic expression of CT166 allows its characterization as a potential virulence factor, as the effect on Ras was more prominent and easier to detect than after infection with *Ctr* D/UW3. In chlamydial infection, CT166 expression and release into the host cell seems to be tightly regulated, and its effects are most likely advantageous for the survival and propagation of *Ctr* D/UW3. Inhibited actin dynamics and migration of infected epithelial cells leads to a loss of epithelial barrier functions, allowing the promotion of chlamydial infection. Furthermore, the effects of CT166 lead to reduced cellular energy usage, because of diminished migration ability, cellular growth and proliferation, or cell cycle progression. The increased energy level could result in improved chlamydial survival because of better supply. Thus, it is not amazing that homologues of CT166 are found in other *Chlamydia* species, including *C. muridarum*, *C. caviae*, *C. psittaci* and *C. felis* [12]. Their characterization is ongoing in our laboratory. Nevertheless, CT166 might act in concert and might share functions with other chlamydial factors. In this regard, CT166 seems to share CT223 functions (such as the capability of inhibiting cell division and inducing multinucleation) [32].

## 3. Experimental Section

### 3.1. Cell Cultures

HeLa cells (Invitrogen, Karlsruhe, Germany) were cultured in Earle’s minimal essential medium (MEM, Biochrom, Berlin Germany) supplemented with 10% fetal calf serum, 2 mM glutamine, 0.1 M nonessential amino acids and 1 mM sodium pyruvate (PAA Laboratories, Pasching, Germany). TRex™-HeLa cells were cultured according to the manufacturer’s instructions in TRex medium (MEM with 10% fetal calf serum and 5 µg/mL blasticidin (Invitrogen)) at 37 °C and 5% CO_2_. Mutagenesis of two aspartic acids of the DXD motif to alanine was carried out using the QuickChange Site-Directed Mutagenesis Kit (Stratagene, La Jolla, CA, USA). The integrity of the constructs was confirmed by sequencing [13]. Clones of HeLa-control, HeLa-CT166-mut and HeLa-CT166-wt were selected in the presence of Zeocin (Invitrogen) [13]. PCR confirmed mycoplasma-free HeLa cells.

### 3.2. Chlamydial Cultures

For stock production, the laboratory-adapted strains, *Chlamydia trachomatis* serovar D/UW3/Cx (ATCC, VR-885) and serovar L2 LGV strain 434 (ATCC, VR-902B), were amplified in HeLa cells. Cell monolayers were infected by centrifugation (55 min, 35 °C, 2000× *g*) in Panserin 401 medium (Cytogen, Berlin, Germany) and 1 µg/mL cycloheximide (Sigma, Deisenhofen, Germany). Infected cells were incubated for 2 days at 37 °C and 5% CO_2_ before EBs were harvested. EBs were released into the supernatant by mechanical destruction of infected cells and centrifugation (15 min, 500× *g*). For the collection of EBs, the supernatant was centrifuged again, this time at 22,000× *g* for 1 h. Afterwards, EBs were washed with transport medium (6.7% saccharose, 40 µg/mL gentamicin, 0.002% phenol red and 2% FCS in PBS) and collected again. For the mock material, HeLa cells were treated identically without chlamydia, but diluted as the infected cells. PCR confirmed mycoplasma-free preparations.

### 3.3. Infection Experiments of TRex™-HeLa Cells

Cells were grown in 6-well plates to sub-confluency. The resulting cell monolayers were washed with PBS and infection with *Ctr* D/UW3 or *Ctr* L_2_/434 (MOI 5 or mock infection) was triggered by centrifugation for 55 min at 35 °C and 2000× *g* in HeLa-TRex medium. After 1 h of incubation at 35 °C and 5% CO_2_, the medium was exchanged. Cells were incubated at 35 °C and 5% CO_2_ during the experiment. Lysates for Western blot were taken 48 h post infection.

### 3.4. Western Blotting

Proteins from cell lysates were separated by SDS-PAGE using 15% polyacrylamide gels according to Maniatis’ manuals [40]. As protein standard, PageRuler™ (#SM0671, Fermentas, Schwerte, Germany) was used. Proteins were transferred to the nitrocellulose membrane for 2 h at 120 V using the Insight Mini Tank Blotter (New England BioGroup, Atkinson, NH, USA). After washing with 0.05% TBST, the membrane was blocked for 1.5 h with 5% nonfat dried milk in TBS containing 0.1% Tween (0.1% TBST). Primary antibodies were applied overnight at 4 °C after additional washing, as follows: rabbit anti-CT166 antiserum, diluted 1:500 in blocking solution [13]; rabbit anti-Ras27H5 (Cell Signaling, Danvers, MA, USA) diluted 1:200; mouse anti-K-Ras F234 (Santa Cruz, Dallas, TX USA) diluted 1:200; rabbit anti-p-p44/42-MAP kinase (Erk1/2) (Cell Signaling) diluted 1:1000; mouse anti-p-pT202/Y204-Erk1/2 (Sigma) diluted 1:1,000; rabbit anti-pT308-Akt Thr 308 (Cell Signaling) diluted 1:1,000; rabbit anti-RhoA 26C5 (Cell Signaling) diluted 1:1000; mouse anti-Rac1 23A8 (Merck Millipore, Billerica, MA, USA) diluted 1:1000; mouse anti-Cdc42 44 (BD Biosciences, Franklin Lakes, NJ, USA) diluted 1:500; mouse anti-βActin (Sigma) diluted 1:5000; mouse anti-chlamydial Hsp60 (Antibodies-online, Aachen, Germany) diluted 1:1000. All antibodies were diluted in 0.05% TBST, unless otherwise stated. After an additional washing step, membranes were incubated at room temperature for 1 h with either anti-rabbit IgG-HRP (GE Healthcare, Chalfont St Giles, UK), diluted 1:1000 in 0.1% TBST containing 5% BSA, or anti-mouse IgG-HRP (MP Biomedicals, Santa Ana, CA, USA), diluted 1:2000 in 0.5% TBST containing 5% non-fat dried milk. The membrane was washed again and developed with SuperSignal West Pico Chemiluminescent Substrate (Thermo Scientific, Waltham, MA, USA). For detection, the Kodak Digital Science Image Station 440 CF (Kodak, Rochester, NY, USA) was used. Quantification was performed using ImageJ software (ImageJ 1.44p, Wayne Rasband, NIH, Bethesda, MD, USA). To calculate the relative intensity of bands, the signal levels of uninfected TRex™-HeLa cells or equally-treated, unaltered TRex™-HeLa cells, respectively, were defined as 1.0 (on the *Y*-axes) for each antibody.

### 3.5. Analysis of Cell Cycle Progression

Equal numbers of HeLa-control, HeLa-CT166-mut and HeLa-CT166-wt were seeded in 6-cm culture dishes and treated with 1 µg/mL tetracycline (Invitrogen) for 24 h. Cells were removed from culture dishes with trypsin/EDTA, washed with PBS and fixed using ice-cold 80% ethanol at −20 °C for 15 min. Upon incubation with RNase (Qiagen, Venlo, The Netherlands) at 37 °C for 30 min, cells were stained with 50 µg/mL propidium iodide (Sigma) at 4 °C for 30 min. Propidium iodide-stained cells were determined by a FACSCalibur cytometer (BD Biosciences) and FlowJo 7.6.5 software (FlowJo LLC, Ashland, OR, USA). Data were presented as histograms for HeLa-control, HeLa-CT166-mut and HeLa-CT166-wt cells representing DNA-dependent fluorescence intensity *versus* frequency within 20,000 analyzed cells. Gates for the determination of the percentages of cells in the 2n, S and 4n stages of the cell cycle were defined in the HeLa-control and then also applied for the two other tested cell lines. The percentage of cells (mean of duplicates) in different stages of the cell cycle was determined in parallel for each cell line (HeLa-control, HeLa-CT166-wt and HeLa-CT166-mut) in three independent experiments. One-way ANOVA and Tukey’s multiple comparison test were used for statistical evaluation.

### 3.6. Cell Proliferation

Equal numbers of cells (HeLa-CT166-mut, HeLa-CT166-wt and HeLa-control) were seeded on 6-well plates for each time point. CT166 expression was induced by adding 1 µg/mL tetracycline. For the determination of the cell number, cells were washed once with PBS, detached by trypsinization (Merck Millipore) and, finally, counted in equal volumes of PBS by using the Scepter™ 2.0 Cell Counter (Merck Millipore).

### 3.7. Quantification of Multinuclear Cell Formation

Cells (HeLa-CT166-mut, HeLa-CT166-wt and HeLa-control) were grown on glass cover slides and treated with 1 µg/mL tetracycline (Invitrogen) for 24h. The cells were washed with 0.3% Triton X-100 in PBS for 5 min and blocked with 5% BSA for about 60 min. Staining of the microtubule network was performed with anti-mouse GTU88 γ-tubulin antibody (Abcam, Cambridge, UK) diluted in PBS containing 3% BSA for 90 min and secondary anti-mouse Alexa 488 conjugated antibody (Invitrogen) diluted in PBS containing 3% BSA. Cells were further stained with DAPI to visualize cell nuclei. Cover slides were mounted with Flouromount G (Southern Biotech, Birmingham, AL, USA). For analysis, Apotome Axio Imager, Axio Vision 4 (Zeiss, Jena, Germany, 2009) and ImageJ software (ImageJ 1.44p) were used.

### 3.8. Scratch Assay

HeLa-CT166-mut, HeLa-CT166-wt and HeLa-control cells were grown to confluent monolayers in 24-well plates and treated with 2 mM thymidine (Sigma) and 1 µg/mL tetracycline. The monolayer was scratched with a 200-µL pipette to create mechanical injury. Afterwards, the medium was exchanged twice to get rid of cell material, and cells were incubated at 37 °C and 5% CO_2_ and analyzed at different time points by phase contrast microscopy (AxioVert 200 M, Kappa Image Base, Zeiss). The coverage of the lesion was determined using T-scratch software [41].

### 3.9. Statistical Analysis

Statistical evaluation of data was performed using either 1-way ANOVA and Tukey’s multiple comparison test, or 2-way ANOVA and the Bonferroni post-test. GraphPad Prism V5 software for Windows (GraphPad Software, Inc., La Jolla, CA, USA) was used for all statistical calculations. Different grades of significance are indicated as follows: * *p* ≤ 0.05; ** *p* ≤ 0.01; *** *p* ≤ 0.001; n.s. = not significant.

## 4. Conclusions

The chlamydial effector protein CT166-wt (not CT166 with mutated DXD (CT166-mut)) causes inactivation of ERK1/2 and Akt, likely through inducing a yet not identified epitope change in Ras-GTPases.

CT166 (not CT166-mut) delays cell proliferation and causes multinucleation during ectopic expression in HeLa cells and might also be causative of the multinucleation observed in chlamydial infection.

Both CT166-wt and CT166-mut inhibit cell migration, suggesting that the mutant cannot be regarded as inactive in any case.
